# A qualitative analysis of negative feelings among incarcerated filicide mothers in Rwanda

**DOI:** 10.1186/s12888-022-04081-0

**Published:** 2022-06-27

**Authors:** Jean d’Amour Muziki, Thaoussi Uwera, Japhet Niyonsenga, Augustin Nshimiyimana, Siméon Gitimbwa Sebatukura, Jean Mutabaruka

**Affiliations:** 1 Department of Clinical Psychology, College of Medicine and Health Sciences, University of Rwanda, Kigali, Rwanda; 2 Department of Health Informatics, College of Medicine and Health Sciences, University of Rwanda, Kigali, Rwanda; 3 Mental Health & Behavior Research Group, College of Medicine and Health Sciences, University of Rwanda, Kigali, Rwanda

**Keywords:** Anxiety, Depression, Anger, Shame, Satisfaction with life, And coping strategies

## Abstract

**Background:**

Most of the research on filicide mothers suggests that they experience negative feelings before they kill their child. However, little is known about whether these negative feelings can be expressed after one-year post-offense among incarcerated filicide mothers with no history of psychiatric problems. In this study, we aimed to conduct a qualitative analysis to (a) understand negative feelings evolving from negative emotions such as anger, guilt, shame, depression, and anxiety among filicide mothers incarcerated in Nyarugenge Prison in Rwanda, (b) identify the impact of experienced negative feelings on their personal wellbeing, and (c) explore their coping strategies.

**Methods:**

This study adopted a phenomenology research design and face-to-face in-depth interviews to explore the problem among twenty filicide mothers selected from Nyarugenge prison. Data were audio recorded, transcribed verbatim, organized, and analysed by using ATLAS.ti 8 Windows.

**Results:**

Anxious and depressed participants experienced both physical and emotional negative feelings. Social withdrawal and cognitive problems were expressed by anxious participants, while avoidance behaviours were particularly experienced by depressed participants.

Intolerance created anger, while self-blame, regret, and acute stress created guilt. In addition, avoidance behaviours and poor self-judgment emerged from shame. Participants felt disconnected from their community and worried about a variety of issues because of their negative feelings. To cope with negative feelings, participants reported that they used abnormal defense, surrender and support from community resources.

**Discussion:**

Our findings highlight the overall negative feelings of incarcerated filicide mothers, which can guide mental health professionals and different stakeholders to respond with appropriate interventions.

**Supplementary Information:**

The online version contains supplementary material available at 10.1186/s12888-022-04081-0.

## Background

Maternal filicide is a social concern due to its detrimental effects on individual, family, and community levels. A filicide mother is defined as a mother who kills her own child aged below 18 years [1, 2]. Despite the introduction of new laws to prevent filicide in late seventeenth century and the adjustment of penalties for those attempting to commit filicide in twentieth century [3], maternal filicide is still a challenge in contemporary society [4] and a leading cause of deaths among children in both underdeveloped and developed countries [5]. Only in developed countries was killing one’s own child reported as the leading cause of child mortality, with rates ranging from 2.4 to 7.0 per 100,000 inhabitants [6, 7]. On a global scale, nearly 95,000 child deaths annually result from filicide [8], with evidence showing that female perpetrators outnumbers male [9].

When collecting data on crimes that come to the attention of the police in Canada, the Uniform Crime Reporting (UCR) Survey showed that 600 cases of homicide are annually identified in Canada and 15% of them are aged under 18 years [10]. Similarly, 1,000 cases of child deaths are annually reported in South Africa, and more than half of these deaths are the result of child abandonment or other parental irresponsibility [11]. Further, homicide rates among children under five years are higher in North America and Sub-Saharan Africa than in higher-income European and Asian countries [12]. Though there is a limited number of studies on maternal filicide in sub-Saharan countries in general and Rwanda in particular, a descriptive study by Mushumba et al. [13] has revealed that 103 cases of filicide were received in 2010 by Legal Medicine, a Department of Kacyiru Hospital. However, this prevalence might be underestimated due to both the moral stigma surrounding it and its perpetration typically being in private, which hinders the obtaining of reliable data on maternal filicide [14].

One of the motives behind maternal filicide is an “*unwanted child*” [15]. Unwanted child filicide stipulates that a child is killed by parents who see the child as unwanted or an obstacle to their freedom [15]. This category of filicide includes parents who benefit from the death of the child in some way (e.g., by inheriting insurance money or marrying a partner who does not want stepchildren) [1]. Other motivating reasons for child filicide include but are not limited to parental intellectual developmental disorder, illegitimacy, and stress associated with social and financial burden [16]. When stress is severe, adults may not be able to regulate their behaviours and negative emotions. Notably, these uncontrolled emotions can turn into destructive, violent behaviour depending on the intensity of the trauma, mental strength, or resilience of the individual [17].

It is well documented that negative emotions such as guilt and shame are experienced by people who have committed violent crimes like maternal filicide [18]. These experiences can be long-lasting and be noticed within three years of the crime [19]. Theoretical evidence has shown that the terms “shame” and “guilt” are clearly different, with significant implications for psychological well-being, even though they are commonly used interchangeably in clinical and more general situations to mean self-conscious cognitive-affective experiences [20, 21]. In this regard, the authors have defined shame as a persistent, uncontrollable psychological state characterized by a generalized negative evaluation of oneself. Through this state, an individual generally experiences feelings of inferiority, powerlessness, and/or vulnerability. Guilt, on the other hand, has been defined as a controllable psychological state associated with a specific behaviour or action that causes remorse or regret [21]. In addition, the authors have highlighted that shame usually leads to feeling bad about “*who you are*” (an intrapersonal cognitive-affective state), while guilt leads to feeling bad about “*what you did to another*” (an interpersonal cognitive-affective state).

Individuals who feel guilty about a violent crime like maternal filicide can display various feelings like regret, remorse, and tension, while those with shame can experience unpleasant feelings aimed towards the self [19]. Some people react to feelings of shame by becoming defensive, denying responsibility, and blaming others [19].

Depression is one of the most cited negative emotions among the cases of filicide mothers [22]. It consists of feelings of intense helplessness or worthlessness, sadness, and hopelessness that persist for several days, weeks, or even months [23]. Key symptoms of depression first include a lack of pleasure, positive reinforcement, or interest in the environment. Other symptoms include anxiety (internal anxiety and panic attacks), decreased emotional involvement (i.e. negative feelings, apathy, and emptiness), boredom and suicidal thoughts followed by increased suicidal risk, lack of concentration or making decisions, inefficiency and passivity, sleep disturbances, and somatic symptoms like fatigue, shortness of breath, and tightness in the chest [24]. Among filicide mothers, the main negative feelings that come from depression are psychotic thoughts or suicidal ideation, a very low mood with crying spells, a lot of worry about the baby’s well-being and the mother’s ability to care for it, irritability, anxiety, insomnia, and fatigue [25].

A previous study has demonstrated that violent crimes (e.g., maternal filicide) and aggression may be the result of high levels of uncontrolled anxiety [26]. This anxiety entails psychological feelings such as “feeling powerless, fearing loss of control, apprehension, stress, worry, and tension as well as physiological changes such as gastrointestinal changes, muscle tension, sweating, tremors, and an increased heart rate and breathing” [27, 28]. High levels of uncontrolled anxiety may also affect the way we feel and behave [29], with evidence showing that women with this anxiety may experience ego-dystonic obsessional thoughts [30].

Uncontrolled anger is another negative emotion in filicide mothers that has gained the attention of researchers [2, 31]. This anger can be defined as a negative, destructive emotion that is frequently associated with writhe, trouble, sorrow, rage [32], a subjective emotional state involving the interaction of cognitive appraisal and psychological components [33], and a state of internal (or phenomenological) negative feeling related to bodily changes, behavioural reactions, and perceptual and cognitive distortions and deficiencies ( i.e. attributing preventability, intentionality, injustice, or blame to others) [34].

Studies have reported that high level of anger and an inability to control are linked to troublesome and destructive behaviours such as violent and aggressive behaviours [35, 36]. In their eight-year retrospective clinical study based on the examination of coroners’ files from Quebec in Canada, Bourget et al. [22] highlighted that retaliating filicide is associated with specific intent to commit murder and can be the result of anger or revenge in a sample of 34 children aged under six years killed by mothers, and 27 filicide mothers. In retaliating filicide, the parent kills the child as a means of exacting revenge upon the spouse, perhaps for infidelity or abandonment. It was also found that outward anger may be characterized by torturing children with verbal or physical abuse and that inward anger may be indicated by suicidal attempts [37].

Most of the qualitative studies conducted in this field have explored perceptions regarding offences, treatment, and rehabilitation processes in South Africa [38], circumstances and characteristics of filicide mothers in England [39], experiences and perceptions of filicide mothers [40], but no studies have investigated negative feelings of incarcerated filicide mothers. A thorough understanding of feelings and their implication for happiness and life satisfaction will serve as a roadmap for the implementation and expansion of interventions aimed at filicide mothers. Therefore, this qualitative study was aimed to explore the negative feelings experienced by incarcerated filicide mothers and the way they affect mothers’ satisfaction with life.

## Methods

### Study design

This study adopted a phenomenological research design to describe negative feelings that filicide mothers had in common. Transcendental phenomenology was chosen as a guiding approach because it prepares professionals for appropriate intervention, explores, and describes the participants’ lived experiences [41]. This approach developed by Husserl [42] consists of three components, namely (1) epoche, (2) noema, and (3) noesis. Epoche refers to the process of discarding preconceived notions and beliefs before conducting phenomenological reflection, and this allows the re-analysation of the phenomenon with a new point of view and different ideas. In this way, all of the parts of a phenomenon come together: noema and noesis [42]. Husserl uses this pair of terms, "Noema" and "Noesis" to refer to correlated elements of the structure of any intentional act. In fact in Ideas, Husserl uses the term ‘Noesis’ to refer to intentional acts or “act-quality” and ‘Noema’ to refer to what, in the Logical Investigations had been referred to as “act-matter”[43]. The current study is the qualitative part of a large study analysis that used a mixed-methods approach to examine the associations between negative emotions and personal well-being among filicide mothers in Rwanda.

### Sample and procedures

The current study used a purposive sample of 20 filicide mothers aged 18 and above who were selected from NYARUGENGE prison to participate in this study. This sample size is in congruence with Creswell’s [44] recommendation that participants should be between five and twenty-five for phenomenological studies. In this study, every participant was called a coresearcher because she expressed negative feelings as her own experience rather than as an investigator’s interpretation of phenomena [44]. Inclusion criteria included having committed maternal filicide, having given birth at least one year ago, and willingness to participate. The individuals were excluded if they had severe physical or mental health problems that would affect their judgement. Co-researchers were recruited in collaboration with a prison social worker.

Ethical approval was obtained from the Institutional Review Board of the University of Rwanda, CMHS (IRB-CMHS). Before starting data collection, we clearly explained the research objectives and research process to the co-researchers. Both verbal and written consent were provided by participants. They were informed about their right to opt out of the study if they didn’t want to participate or if they changed their mind. After completion of questionnaires for the quantitative phase, 20 co-researchers were invited and accepted to participate in this qualitative study after a one-week period. The participants expressed their lived negative feelings layered with anger, anxiety, depression, guilt, shame, or lower level of happiness and satisfaction with life. In-depth individual interviews were conducted in Kinyarwanda in safe and comfortable places. One research assistant with a master’s degree in Clinical Psychology supported the conducting and recording of the interviews. This person has hands-on experience of conducting qualitative research interviews and working with distressed mothers.

### Data collection

Qualitative data was collected using a semi-structured interview guide that was developed based on study objectives. Sample questions were: (1) Have you been anxious after committing maternal filicide? (2) Have you ever felt depressed after committing maternal filicide? (3) Have you ever felt guilt after committing maternal filicide? The present study adopted semi-structured interviews for the deep exploration of various negative feelings experienced after committing maternal filicide by asking more open-ended questions. Consistently, semi-structured interviews were found to be an effective method for data collection when the researcher wanted to: (1) collect qualitative, open-ended data; (2) explore participant thoughts, feelings, and beliefs about a particular topic; and (3) delve deeply into personal and sometimes sensitive issues [45]. Furthermore, probing questions were used to gather more detailed information on experienced negative feelings. For instance, can you explain more about how you felt? Could you give me an example of the recurrent feelings? Interviews lasted between 40 and 60 min.

### Data analysis

This qualitative data was audio recorded and transcribed verbatim, which was then analysed following three main steps: (1) horizontalization, (2) imaginative variation, and (3) synthesis. In the early first step, a team of five researchers coded statements from transcripts that included details about the phenomenon under investigation. The second step was imaginative variation, where they defined the phenomenon’s importance, which guided them in deriving structural themes, and the last step was synthesis that reflects the meanings and essence [42].

During the data analysis, the team of five researchers sat together to analyse and code the first interview. For the quotes where the coding was not uniform for all researchers, the discrepancies were discussed and removed. The same process was applied to the second, the third, and the fourth interview. The remaining sixteen interviews were then allocated to four researchers, and they met after coding to agree on new codes and deliberate on themes that reflect experiences of filicide mothers. An inductive coding style was applied to our data, which helped to create as many codes as possible. Thereafter, important codes were grouped to form categories (themes), which were later incorporated into results and data analysis. Later, a connection among the themes was described. ATLAS.ti 8 Windows was used to organize, store, and retrieve information clearly in the process of data analysis and let it be available easily later. As a result, we could now understand how filicide mothers develop and express negative emotions and feelings differently.

## Results

### Sociodemographic characteristics of the participants

Table 1 summarises the sociodemographic characteristics of the study participants. The sample was composed of 20 filicide mothers aged 23 to 45 years (mean age = 29 years, *SD* = 7.36) who participated in this qualitative study. Many respondents were aged from 25 to 29 years (7/20), followed by those aged less than 25 years (6/20), and the rest were aged from 30 to 34 years (3/20), 35 to 39 years (3/20), and 45 years (1/20). Most of the study participants had been incarcerated for 2 to 3 years (12/20), followed by 1 year (6/20), and the least had been incarcerated for at least 4 years (2/20). Almost half of the respondents were primiparous (9/20) and the rest were multiparous (11/20). The sample was predominantly composed of single mothers (16/20) at the time of committing the crimes (Table 1).

**Table 1:** Participants’ sociodemographic characteristics (*n* = 20)

**Age category**	**n**	**Number of children **	**n**
Under 25	6	None	9
25-29	7	One child	5
30-34	3	Two children	4
35-39	3	More than two children	2
45	1		
**Years spent in prison**		**Marital status**	
1	6	Single	16
2-3	12	Separated	4
4 and above	2		

## Structural description

As presented in table 2, the analysis resulted in seven themes that represented feelings of anxiousness, feelings of depression, feelings of anger, feelings of shame, feelings of guilt, impact on happiness and satisfaction with life, mechanisms for management of negative feelings for negative feelings among incarcerated filicide mothers that emerged with several subthemes.

**Table 2:** Summary of the main themes and sub-themes

Main themes	**Sub-themes**
3.3. Feelings of anxiousness	3.3.1. Emotional changes
	3.3.2. Bodily changes
	3.3.3. Cognitive problems
	3.3.4. Social withdrawal
3.4. Feelings of depression	3.4.1. Bodily responses
	3.4.2. Emotional responses
	3.4.3. Avoidance behaviours
3.5. Feelings of anger	Intolerance
3.6. Feelings of shame	Avoidance behaviours and poor self-judgment
3.7. Feelings of guilt	Acute stress, regret, and self-blame
3.8. Impact on happiness and satisfaction with life	3.8.1. Lack of the link with the community.
	3.8.2. Mixture of worries
3.9. Mechanisms for management of negative feelings	3.9.1. Abnormal defense
	3.9.2. Surrender
	3.9.3. Support from community resources

### Feelings of anxiousness in filicide mothers

The feelings of anxiousness emerged with four main subthemes: emotional changes, bodily changes, cognitive problems, and social withdrawal.

#### Emotional changes

Many respondents expressed emotional changes in three categories: “feeling lonely,” “rumination and tears,” and, “hatred and worthlessness,” as well as “suicidal ideations.”

* “I looked like someone who lived alone without parental care because my parents were busy drinking beer, getting drunk and getting wasted”-P9.*

*“Simply I thought about myself! I had failed to accept myself, and when I passed by people, I thought they were talking about me; no one actually spoke to me nicely in the village. It was only tears for me”-P20.*

*“I started feeling that I had become like a patient with mental disorders, I started acting without clothing and cried loudly, having become like a mentally patient” -P19.*

#### Bodily changes

The participants reported the experience of physiological feelings such as “sleep disturbances”, “trouble breathing and tears”, “fatigue and irritability”, “headaches”, “weight loss”, and loss of appetite.

*“I have been extremely anxious. I am pregnant without a husband; I will therefore give birth to a child with no address! I will be insulted, I will be ashamed, I will find nowhere to go, I will find no stability, and it is all these things that make me anxious. I suffered from insomnia, and I always felt bad!”-P12.*

*“I had trouble breathing, then I heavily shed tears, my eyes became red. I can’t really know how to tell you.” -P19.*

#### Cognitive problems

Cognitive feelings such as ‘losing concentration’ were also reported.

“*During my studies, I could memorize nearly 10 papers, but after that, I could only read one paper and felt unable to continue.* –P1”.

#### Social withdrawal

Most of the respondents argued social withdrawal feelings were represented by a lack of pleasure in social situations and a low interest in peer convivial gatherings.

*“Before, I was very sharp, but after committing a crime, I started being very shy because of many thoughts. I refrained from interacting with people with whom we used to socialize, thinking that they already knew mine. I sat alone, isolating myself” -P1.*

### Feelings of depression among filicide mothers

With regard to the feelings of depression, three subthemes were found: bodily responses, emotional responses, and avoidance behaviours.

#### Bodily responses

The weight loss” and “loss of appetite” were argued by respondents. Here are the verbatim quotes.

*“One of the things that indicated that I was depressed is that I became too thin” -P3.*

*“I could not eat as usual; I ate too little. I picked food once from a dish and I fail. I did not even like to eat” -P3.*

#### Emotional responses

Emotional responses were emerged in seven categories: “frequent tears and getting annoyed quickly”, “suicidal ideations”, “fear of death”, “feeling lonely”, “losing the sense of inner humanity”, “inability to talk”, and situational avoidance. Here are the sample quotes:

*“The symptoms of my depression included crying; I couldn’t talk well”-P19.*

*“I felt excessively depressed up until I wanted to commit suicide, but I thought I would grieve my child. Wherever I stayed alone I felt a social withdrawal, I was so anxious that I wished I would even die.”-P19.*

*“I did not feel inner humanity, I did not talk, you could hurt me, and I look at you without talking. I shed tears abundantly and I suffered a chronic headache, an everyday headache. I got medical treatment, they screened me but in vain” -P19.*

### Avoidance behaviours

Almost a half of respondents reported situational avoidance like avoiding usual groups, relationship with males, family interactions and other social interaction events.

*“I felt that no one might approach me; I did not want anyone to come and visit me!” -P12.*

### Feelings of anger among filicide mothers

#### Intolerance

Intolerance was clustered into “avoiding physical contact”, “no tolerance to frustration”, and “inability to talk”.

*“I did not want anyone to approach me, it was special for children because I did not want their disturbance”-P19.*

*“When you passed by me and step on me, I immediately insulted you”-P7.*

*“If for example, a cup or a small jerrycan were fallen down by a child, I felt irritable, in case he/she does not go away from me, I felt I could even beat him. I could pass by you and you don’t hear when I greet you, then I feel very anger or once I pass by people and I hear they are talking I guess it is I whom they are talking about then I feel anger. I really felt anger about both invisible and visible things! When you really face the same problem I faced, it is like you have lost humanity, you have just entered in another special person who does not exist”–P12.*

*“Symptoms I display are that it becomes too difficult for me to talk to anyone or if someone talks to me, I reply negatively by insulting him”-P14.*

### Feelings of shame among participants

#### Avoidance behaviours and poor self-judgment

Avoidance behaviours and poor self-judgment’ was represented by various experiences such as “avoiding eye contact”**, “**irrational prejudice”, “avoiding crime related conversations”, **“**fear of gossip from the community”, and inability to talk in the public.

*“Sometimes I looked at people and I felt I don’t want even to face them. I felt I would rather walk looking at down” –P10.*

*“You felt that everyone who will see you will at once recognize the crime you have committed. When you pass by someone for example talking freely about other kind of stories you think he is talking about you, then I ask him ‘What are you talking about me?’ You really feel like what you have done is written on your face so that each and every one is looking at it” -P13.*

*“When people are talking about the crime I committed, I don’t even want to stay near to them, and I prefer staying far from them” -P2.*

The participants also expressed fear of gossip from the community and inability to talk in the public during the interview.

*“Shame is surely there, I really ask myself, will I go to my village if I go back home? Where will I go? You really feel ashamed! You can go home coming from somewhere, you pass by someone and then they say she has killed her child, what reason did push her to kill him? They just criticize you through gestures and you find nothing to say” –P4.*

*“I felt I don’t want to talk while sitting together with people because of shame” –P10.*

### Feelings of guilt among participants

#### Acute stress, regret, and self-blame

Acute stress, regret and self-blame emerged with three experiences: self-blame, a regret for the deceased child, and heartbeat and fear**.**

*“Even now I feel guilty. I feel that I didn’t do good things but, unfortunately nothing else I can do!” –P7.*

*“Sometimes I looked at child coming towards me, and I felt I can’t really hold him for I felt guilty and then I stated ‘If I did not kill mine, he would be of the same age like this one’” –P10.*

*“Yes, I always felt a high heartbeat, having culpability and fear. Time came and I felt no peace, no how to behave, whenever I went to bed, I really felt no peace and I felt extreme fear” –P14.*

### Impact of experienced negative feelings on happiness and satisfaction with life

Impact of experienced negative feelings on happiness and satisfaction with life emerged with two subthemes: “lack of the link with the community” and “mixture of worries”.

#### Lack of the link with the community

Feeling rejected by the community, feeling of left behind and feelings of worthlessness were frequent impact that was reported by many participants. Filicide mothers mentioned that family members stopped giving them love and attention hence they felt progressively low satisfaction about life:

*“Imagine really, oh my God, I have been extremely dissatisfied. Only God who knows! I was hated by both my family and brothers and sisters; they did not want to visit me and even my friend who impregnated me rejected me” –P7.*

*“It has been too difficult for me, look at the years I have spent in prison and look at how old I am today, you will actually say that I lagged behind because of history. In all years I spent being jailed, I would have reached the following: I would have completed my studies, I would have got a job, I would have married, I would have attained certain things”–P19.*

*“I felt no one can love me again, and actually felt I was no longer needed.”–P7.*

#### Mixture of worries

Some participants were worried about the source of basic human needs, and social and judicial services-related worries and worries about future.

*“You see others have got cars, they have got money, they have a job, all these make one ask herself ‘Oh, my God, why did this happen to me’ I do not have any parent, I do not possess anything either, even if someone harasses me, I can’t be able to defeat him! You actually feel no happiness!” –P13.*

*“I have been greatly dissatisfied with life, very much indeed. Poverty, eating irregularly, eating what you don’t want, queuing, lack of freedom”.P14.*

*“If I think about the life in which I was living, that I was not poor! And I think about a way I used to develop myself, that I begged somewhere when life was difficult for me. And also, I think how I was going back below zero, and then I felt life is over.”-P7.*

### Mechanisms for management of experienced negative feelings

Abnormal defense, surrender, and support from community resources are three subthemes that were formed to show strategies employed by filicide mothers to manage experienced negative feelings after committing maternal filicide.

#### Abnormal defense

Some participant argued “avoidance of thoughts linked to committed crime” and “inability to overcome difficulties and suicidal ideations” to deal with the emotions felt.

*“Yes, I tried to deal with those feelings but sometimes it happens to be difficult, as for me, I avoid thinking more about it” –P1.*

*“Till now I have never tried to deal with them, personally the problem is that they keep an eye on us because there are people with life imprisonment, and some are hopeless. On my opinion, I wish I would go where multistorey buildings are, because we have them here, I feel I would fall down from a storied house and die”-P2.*

*“I never tried it and was unable because I felt that I had no more strength! I was always crying I had put my telephone offline; there was no other way of dealing with them! I waited for the death and in vain” –P7.*

*“How can I then tell you the way I tried to deal with them as long as you have heard the decision I made? No attempt to deal with them actually, as long as it was beyond my control.” -P12.*

#### Surrender

Surrender is a theme that is clustered by three strategies. First of all, there were participants who noted that “*patience*” helped them to survive.

*“I tried to be patient with life, I tried to value myself, I applied avoidance and not indulging in immoral lifestyle” -P13.*

*“I tried to deal with them, I accepted them to be mine because I would go nowhere else”-P14.*

The respondents also preferred to calm down and surrender and going to a lonely place”.

*“Of course I calmed down at once and try to put them out of my mind” -P11.*

*“If you do not want to show people that it did not make you sad because they often laugh at you wondering why you did not deliver, I simply show them that It did not sadden me as a way of avoiding them, then I go to bed and cover myself with a blanket, I shed tears and it finally stops.” -P1.*

#### Support from community resources

Support from community resources is a theme that encompass normal strategies that were applied by the participant to deal with experienced negative feelings. *Relying on prayers*” was the most cited strategy. “

*Praying and presenting this to GOD is one of the things that maybe helps me feel there is another life after this, I tell God, ‘All my problems are presented to you who will do something on them and put an end to them.” –P3.*

*“We actually can do all things through Christ who strengthens us. If you are capable of praying, first of all, you accept yourself, when you have accepted yourself you also accept what happened to you, finally, you get hope for the future.”–P19.*

Having a good company, joining social groups, and being imprisoned are other strategies that helped them to manage the experienced negative feelings.

*“You see, I got out of all these when I reached in prison, I really find that going to prison is what saved me, it is what saved my heart! The reason is that I got a time to repent the crime I committed. I saw a time to accept it, to repent and ask for forgiveness. But if it were not known that I be taken and jailed, I would not be still alive! I wouldn’t really be still alive! Getting someone who will listen to you and who will not go and talk about you everywhere, rather, he keeps a secret about you, well and good, this helps you to be free.”-P12.*

## Discussion

Our study explored negative feelings embedded in negative emotions like anger, depression, shame, guilt, and dissatisfaction with life among incarcerated filicide mothers in Rwanda. The current study revealed that feelings of anger were clustered into one subtheme: “intolerance.” This subtheme was reflected in a variety of experiences, including aversion to physical contact, inability to communicate, and a sensitivity to frustration. Worryingly, authors have indicated that causation of other people’s harm and a higher level of self-destruction were found in the sample of people who had experienced violent anger [46]. Furthermore, people who experience feelings of anger and stressful life events tend to develop negative feelings of lower self-esteem and depression. Besides, both feelings of anger and stressful life events play an important role in depression assessment [47]. Consistently, it has been found that filicide mothers suffer from negative feelings of anger [48].

Our findings indicate the negative feelings related to depression are evident in the current sample. Similarly, a prior study have suggested that filicide mothers have frequent negative feelings of depression, such as suicidal ideation or even psychotic thoughts, severely depressed mood with crying spells, preoccupation with worries about the baby’s well-being and the mother’s caring abilities, irritability, insomnia, fatigue, and anxiety [25]. In our study, depression consisted of physical experiences like weight loss, emotional experiences (e.g., fear of death, feeling lonely, losing the sense of inner humanity, suicidal ideations, frequent tears, and getting annoyed quickly), and avoiding behavioural experiences like situational avoidance. Our findings are supported by the study conducted on a sample of ten Finnish mothers who had unintentionally killed their children [25]. However, avoidance behaviours tending to situational avoidance, including avoiding relationships with males, peer groups, family interactions, and other social interaction events, were particularly observed in our study. One study showed that the onset of feelings of depression leads to unhealthy parent behaviours and poor interpersonal reactions [49]. Other studies [50, 51] found that negative feelings of depression and anxiety co-occur. In addition, a close connection was found between negative feelings of anxiety and depression [52].

Taking account of the foregoing, it has been found that filicide mothers suffer from negative feelings of anxiety [25]. For the participants, anxiety was composed of emotional experiences (e.g., feeling angry, feelings of hatred, feeling lonely, feelings of worthlessness, lack of inner peace, losing one’s mind, remaining silent, suicidal ideations, rumination, and cries), bodily experiences (e.g., weight loss, headaches, loss of appetite, trouble breathing, sleep disturbances, fatigue, and irritability), cognitive problems like losing concentration and social withdrawal experiences like low interest in peer convivial gatherings. These experiences are similar to those highlighted by prior studies [53–55]. Conversely, symptoms of anxiety such as frustration, guilt, feelings of loss [54], challenging self-treatment (e.g., self-criticism, self-interruption, and worry), apprehension (chronic painful emotions and fear of triggers), emotional avoidance (e.g., self-distraction), behavioural avoidance (e.g., over-compliance and avoidance of conflict) and unmet needs (e.g., to be protected, to be loved, to be acknowledged) [53] were not found in the current study. In the same vein, an increasing number of currently conducted studies show that shame and guilt are significant features of different psychopathological conditions, including anxiety [56].

As argued by the participants, shame consisted of avoidance behaviours and poor self-judgment experiences that emerged with subthemes such as irrational prejudice, avoidance of crime-related conversations, avoidance of eye contact, inability to talk in public, and fear of gossip from the community. Even though little is known about the feelings of shame in filicide mothers, one study highlighted significant experiences, especially among women victims of intimate partner violence. They are, for instance, creating the perfect illusion of isolation, selfhood, and marginalized identities [57]. However, they are totally different from the ones displayed by our participants. Another study showed that shame is a leading cause of all violence, either toward the self or others [58]. However, this violence has the purpose of reducing the degree of shame experienced by an individual and replacing it with its opposite (pride), and this prevents the individual from being gnawed by the feelings of shame [58]. Similarly, the co-occurrence of feelings of shame and guilt was found in various samples [59, 60].

Guilt takes place when a suspected person agrees to take part in immoral action that leads to harming another person [61]**.** Guilt is a motivational state like other emotions and plays a large part in promoting reparative actions like compensating for a harmful behaviours or apologizing [62]. Based on interviews with our participants, symptoms of guilt such as self-blame, acute stress (heartbeat and fear) and regret (regret for the deceased child) were heard. The guilt that was found in the interviewed filicide mothers could help them learn from their experiences. As a result, they will be able to avoid similar violent acts in the future [63].

Furthermore, negative emotions and mental distress were found to be significant predictors of life dissatisfaction [64]. For our participants’ perspectives, life dissatisfaction is associated with a lack of link with the community (e.g., feeling rejected by the community, feeling left behind, and feelings of worthlessness) and a mixture of worries (e.g., worries about the future and basic human needs, and social and judicial service-related worries). In congruence with our findings, it was found that living alone, poor social support, and unsustainable income can increase the degree of life dissatisfaction in our participants [64]. Long-term life dissatisfaction raises the risk of developing various mental disorders, including depression [64].

Filicide mothers express several times their deep regret for what happened. They always ask themselves how they will be trusted again, who will collaborate with them again, who will hear their problems, etc. They think about themselves, they recall what happened to them, and they feel unable to endure. Despite the mentioned challenges, some participants stated that support from community resources (e.g., a good company, the existence of a prison, social groups, and prayers) helped them to manage the situation. One study notes spiritual dimensions correlate negatively with mental distress, which means the more spiritual dimensions increase, the more mental distress symptoms decrease [65]. Another study reveals that spiritual-religious psychotherapy has the ability to reduce feelings of depression, anxiety, and stress [66].

In addition, it has been proven that education of peer workers, home visits, organization of support groups, and provision of psychological support lead to the recovery process of people with mental disorders [67]. While some participants use community support to cope with negative feelings, others rely on abnormal defences such as avoidance behaviours and suicidal ideation, or surrender behaviours such as loneliness. Avoidance behaviours are measures taken by individual to avoid unpleasant feelings and thoughts [68], suicidal ideations are feelings and thoughts of self-harm [69], surrender behaviours are beliefs in which participants believe that experienced negative feelings and thoughts are beyond human control [70], while loneliness is an unpleasant feeling brought on by a lack of meaningful connections[71]. In a sense, a filicide mother protects herself from painful feeling by avoiding any reminder of what happened, thinking about killing herself, allowing herself to be controlled by another power, and staying alone.

## Study strengths and limitations

The strength of this study is that it was conducted in one of the sub-Saharan countries where there is a dearth of studies on maternal filicide in general and the use of qualitative analysis in particular to explore the experiences of filicide mothers after committing maternal filicide. The findings showed that negative feelings among participants are intense one year after committing the crime. This implies that filicide mothers are more vulnerable and that regular screening for mental health issues is needed for appropriate interventions. Findings also highlighted that prevention and reintegration strategies should be used in different parts of Rwanda to make mothers at risk of maternal filicide and filicide mothers valuable in their own communities.

Several limitations should be considered vis-à-vis the study findings. Our study was a qualitative analysis that used a transcendental phenomenological research design and face-to-face in-depth interviews. This means that the sample was small and there is no possibility to generalize the findings to a larger sample. In addition, qualitative studies regarding negative feelings post-maternal filicide are sparse in the literature, especially in sub-Saharan countries. Therefore, it was not easy to find scientific evidence to support our study conclusions. In this regard, more studies on negative feelings for women who committed filicide and were found responsible for this crime are needed for further management. Finally, the sample was institutional-based (prison) and we did not assess the effect of imprisonment on experienced negative feelings. It is possible that imprisonment might have worsened the level of negative emotions experienced. Therefore, future studies should compare these negative feelings between incarcerated filicide mothers and those who are in the community (i.e., the ones released by the presidential grace) to improve the conclusion and clinical practice.

## Implication for practice

The study findings provide a general picture of experienced negative feelings that can guide mental health practitioners and different stakeholders through appropriate rehabilitation and reintegration of filicide mothers. One study revealed that the successful elimination of maternal filicide should be preceded by a good understanding of the lives of filicide mothers [72]. As such, healing maternal filicide related wounds and assessing their day-to-day lives will have a positive impact on both rehabilitation and reintegration.

## Supplementary Information

**Additional file 1.**

## Data Availability

Data analyses performed during the current study (recordings and transcripts) are available from the corresponding authors on reasonable request.

## References

[1] West SG (2007). An overview of filicide. Psychiatry (Edgmont).

[2] Krischer MK, Stone MH, Sevecke K, Steinmeyer EM (2007). Motives for maternal filicide: results from a study with female forensic patients. Int J Law Psychiatry.

[3] Tang D, Siu B (2018). Maternal infanticide and filicide in a psychiatric custodial institution in Hong Kong. East Asian Arch Psychiatry.

[4] Putkonen H, Amon S, Weizmann-Henelius G, Pankakoski M, Eronen M, Almiron MP (2016). Classifying filicide. Int J Forensic Ment Health.

[5] Eke SM, Basoglu S, Bakar B, Oral G (2015). Maternal filicide in Turkey. J Forensic Sci.

[6] Barone L, Carone N (2021). Childhood abuse and neglect experiences, hostile-helpless attachment, and reflective functioning in mentally ill filicidal mothers. Attach Hum Dev.

[7] Flynn SM, Shaw JJ, Abel KM (2013). Filicide: mental illness in those who kill their children. PLoS One.

[8] Unicef. Hidden in plain sight: a statistical analysis of violence against children. UNICEF: New York; 2014.

[9] Putkonen H, Weizmann-Henelius G, Collander J, Santtila P, Eronen M (2007). Neonaticides may be more preventable and heterogeneous than previously thought - neonaticides in Finland 1980–2000. Arch Womens Ment Health.

[10] Kong R. Statistiques de la criminalité au Canada, 1996. Ottawa: Juristat; 1996.

[11] Mathews S, Abrahams N, Jewkes R, Martin L (2013). Underreporting child abuse deaths: experiences from a national study on child homicide. S Afr Med J.

[12] Putkonen H, Amon S, Almiron MP, Cederwall JY, Eronen M, Klier C (2009). Filicide in Austria and Finland - a register-based study on all filicide cases in Austria and Finland 1995–2005. BMC Psychiatry.

[13] Mushumba H, Hakizimana FX, Murangira TB, Nyamwasa D, Schroeder AS, Sperhake J (2016). Trends and patterns of suspected infanticide cases autopsied at the kacyiru hospital, Rwanda: case report. Rwanda Med J.

[14] Emery JL (1993). Abuse, sudden infant death syndrome, and infant death. Ajdc.

[15] Resnick P (2016). Filicide in the United States. Indian J Psychiatry.

[16] Mugavin ME (2005). A meta-synthesis of filicide classification systems: psychosocial and psychodynamic issues in women who kill their children. J Forensic Nurs.

[17] Spinelli MG (2010). Denial of pregnancy: a psychodynamic paradigm. J Am Acad Psychoanal Dyn Psychiatry.

[18] Riaz M (2018). Exploring guilt and shame among violent criminals. Sociol Criminol Access.

[19] Tangney JP, et al. Two faces of shame: the roles of shame and guilt in predicting recidivism. Psychol Sci. 2014;25(3):799–805. 10.1177/0956797613508790. 10.1177/0956797613508790 PMC4105017 24395738

[20] Bryan CJ, Morrow CE, Etienne N, Ray-Sannerud B (2013). Guilt, shame, and suicidal ideation in a military outpatient clinical sample. Depress Anxiety.

[21] Kim S, Thibodeau R, Jorgensen RS (2011). Shame, guilt, and depressive symptoms: a meta-analytic review. Psychol Bull.

[22] Bourget D, Grace J, Whitehurst L (2007). A review of maternal and paternal filicide. J Am Acad Psychiatry Law.

[23] Foreman R (2015). Depression self help: how to deal with depression, overcome depression and symptoms and signs of depression richard foreman.

[24] Djukanovic I. Depression in older people. 2017;38.

[25] Kauppi A, Kumpulainen K, Vanamo T, Merikanto J, Karkola K (2008). Maternal depression and filicide - case study of ten mothers. Arch Womens Ment Health.

[26] Neumann ID, Veenema AH, Beiderbeck DI (2010). Aggression and anxiety: social context and neurobiological links. Front Behav Neurosci.

[27] Stark D, Kiely M, Smith A, Velikova G, House A, Selby P (2002). Anxiety disorders in cancer patients: their nature, associations, and relation to quality of life. J Clin Oncol.

[28] Kazdin A (2011). Encyclopedia of psychology: 8 volume set. Am Psychol Assoc.

[29] Baqutayan SMS (2012). The effect of anxiety on breast cancer patients. Indian J Psychol Med.

[30] Spinelli MG (2004). Maternal infanticide associated with mental illness: prevention and the promise of saved lives. Am J Psychiatry.

[31] Lewis CF, Bunce SC (2003). Filicidal mothers and the impact of psychosis on maternal filicide. J Am Acad Psychiatry Law.

[32] Modrcin-McCarthy MA, Pullen L, Barnes AF, Alpert J (1998). Childhood anger: so common, yet so misunderstood. J Child Adolesc Psychiatr Nurs.

[33] Nasir R, Ghani NA (2014). Behavioral and emotional effects of anger expression and anger management among adolescents. Procedia - Soc Behav Sci.

[34] Kassinove H, Sukhodolsky DG. Anger disorders: Basic science and practice issues. Anger Disord Defin Diagnosis, Treat 2014:1–27. 10.4324/9781315820361.

[35] Blair RJR (2012). Considering anger from a cognitive neuroscience perspective. Wiley Interdiscip Rev Cogn Sci.

[36] Levinson MH, Share P, City NY (2006). Anger management and violence prevention. A Rev Gen Semant.

[37] Shouse JL (2013). Behavioral characteristics of maternal filicide: a case study.

[38] Sanushka M. Qualitative study of mentally Ill women who commit Filicide in Gauteng, South Africa. Front Psychiatry. 2019;10 757. 10.3389/fpsyt.2019.00757. 10.3389/fpsyt.2019.00757 PMC6821781 31708816

[39] Sidebotham P, Retzer A. Maternal filicide in a cohort of English Serious Case Reviews. Arch Womens Ment Health. 2019;22(1):139–49. 10.1007/s00737-018-0820-7. 10.1007/s00737-018-0820-7 PMC6373272 29500658

[40] Milia G, Noonan M. Experiences and perspectives of women who have committed neonaticide, infanticide and filicide: a systematic review and qualitative evidence synthesis. J Psychiatr Ment Health Nurs 2022:1–16. 10.1111/jpm.12828. 10.1111/jpm.12828 PMC9790608 35255182

[41] Murguia E, Díaz K (2015). The philosophical foundations of cognitive behavioral therapy: stoicism, buddhism, taoism, and existentialism. J Evidence-Based Psychother.

[42] Moustakas CE. Phenomenological research methods. New York; 1994.

[43] Rassi F, Shahabi Z (2015). Husserl’s phenomenology and two terms of noema and noesis. Int Lett Soc Humanist Sci.

[44] Creswell JW, Hanson WE, Clark Plano VL, Morales A (2007). Qualitative research designs: selection and implementation. Couns Psychol.

[45] DeJonckheere M, Vaughn LM (2019). Semistructured interviewing in primary care research: a balance of relationship and rigour. Fam Med Community Heal.

[46] Allen D, Bethell K, Allen-Carroll M (2016). Anger and social fragmentation: the evil violence tunnel. J Psychother Integr.

[47] Busch FN (2009). Anger and depression. Adv Psychiatr Treat.

[48] Carruthers G (2016). Making sense of spousal revenge filicide. Aggress Violent Behav.

[49] Paulson JF, Dauber S, Leiferman JA (2006). Individual and combined effects of postpartum depression in mothers and fathers on parenting behavior. Pediatrics.

[50] Alansari B (2005). Relationship between depression and anxiety among undergraduate. Soc Behav Pers.

[51] Huffman J (2010). The relationship between depression, anxiety, and cardiovascular outcomes in patients with acute coronary syndromes. Neuropsychiatr Dis Treat.

[52] Van Oers HA, Haverman L, Limperg PF, Van Dijk-Lokkart EM, Maurice-Stam H, Grootenhuis MA (2014). Anxiety and depression in mothers and fathers of a chronically Ill child. Matern Child Health J.

[53] Brien KO, Keeffe NO, Cullen H, Durcan A, Timulak L, Mcelvaney J, et al. Emotion-focused perspective on generalized anxiety disorder : a qualitative analysis of clients ’ in-session presentations qualitative analysis of clients ’ in-session presentations. 2017;3307. 10.1080/10503307.2017.1373206.

[54] Ali E (2018). Women’s experiences with postpartum anxiety disorders: a narrative literature review. Int J Womens Health.

[55] Mason JE, Faller YN, LeBouthillier DM, Asmundson GJG (2019). Exercise anxiety: a qualitative analysis of the barriers, facilitators, and psychological processes underlying exercise participation for people with anxiety-related disorders. Ment Health Phys Act.

[56] Cândea DM, Szentagotai-Tăta A (2018). Shame-proneness, guilt-proneness and anxiety symptoms: a meta-analysis. J Anxiety Disord.

[57] Thaggard S, Montayre J (2019). “There was no-one I could turn to because I was ashamed”: shame in the narratives of women affected by IPV. Womens Stud Int Forum.

[58] Sampson R, Tonry M, Anderson E, Ogletree C, Tyler T, Simon J (2001). Crime , Inequality and the State.

[59] Harder DW, Cutler L, Rockart L (1992). Assessment of shame and guilt and their relationships to psychopathology. J Pers Assess.

[60] Blythin SPM, Nicholson HL, Macintyre VG, Dickson JM, Fox JRE, Taylor PJ (2020). Experiences of shame and guilt in anorexia and bulimia nervosa: a systematic review. Psychol Psychother Theory, Res Pract.

[61] Michalos AC. Encyclopedia of quality of life and well-being research. 2014. 10.1007/978-94-007-0753-5

[62] Lerner JS, Li Y, Valdesolo P, Kassam KS (2015). Emotion and decision making. Annu Rev Psychol.

[63] Herek GM, McLemore KA (2013). Sexual prejudice. Annu Rev Psychol.

[64] Rissanen T, Viinamäki H, Honkalampi K, Lehto SM, Hintikka J, Saharinen T (2011). Long term life dissatisfaction and subsequent major depressive disorder and poor mental health. BMC Psychiatry.

[65] Bonab BG, Hakimirad, Habibi. Relation between mental health and spirituality in Tehran University student. Procedia - Soc Behav Sci 2010;5:887–91. 10.1016/j.sbspro.2010.07.204.

[66] SedaghatGhotbabadi S, Haji Alizadeh K (2018). The effectiveness of spiritual-religion psychotherapy on mental distress (depression, anxiety and stress) in the elderly living in nursing homes. Heal Spiritual Med Ethics.

[67] D Stimac Grbic, I Pavic Simetin AI. The importance of peer support in the recovery process of persons with mental disorders. Eur J Public Heal. 2020;30. 10.1093/eurpub/ckaa166.1025.

[68] Krieglmeyer R, De Houwer J, Deutsch R (2013). On the nature of automatically triggered approach-avoidance behavior. Emot Rev.

[69] McAuliffe CM (2002). Suicidal ideation as an articulation of intent: a focus for suicide prevention?. Arch Suicide Res.

[70] Duckham BC, Greenfield MJ. Surrender and contemporary social work theory. Crit Soc Work 2019;10. 10.22329/csw.v10i1.5795.

[71] Huang YJ, Wang KY, Chen CM (2010). Loneliness: a concept analysis. J Nurs.

[72] Oberman M (2003). Mothers who kill: cross¬cultural patterns in and perspectives on contemporary maternal filicide. Int J Law Psychiatry.

