# Supplementary material for: A qualitative analysis of negative feelings among incarcerated filicide mothers in Rwanda
Source: BMC Psychiatry. 2022 Jun 27;22:432. doi: 10.1186/s12888-022-04081-0 (PMC9235198; doi:10.1186/s12888-022-04081-0)
Supplement: Supplementary file 1 — Additional file 1. [file 12888_2022_4081_MOESM1_ESM.docx]

**Appendix**

**Semi-structured interview guide**

(1) Have you been anxious after committing maternal filicide? If yes, explain how you have felt. What are the symptoms of anxiety that you have felt?

(2) Have you ever felt depressed after committing maternal filicide? If yes, how have you felt? Tell us about the elements that came the most?

(3) Have you ever felt angry after committing maternal filicide? If yes, what was it like? How have you felt?

(4) Have you ever been ashamed after committing maternal filicide? If yes, how have you felt? Can you give us some examples of how you have felt?

(5) Have you ever felt guilt after committing maternal filicide? If yes, how did you feel? What were predominant feelings?

(6) Have you ever been dissatisfied with the quality of life after committing maternal filicide? If so, what were the recurrent feelings?

(7) How did you manage negative feelings after committing maternal filicide? Give examples of strategies that you used.
